# Identifying the Deleterious Effect of Rare *LHX4* Allelic Variants, a Challenging Issue

**DOI:** 10.1371/journal.pone.0126648

**Published:** 2015-05-08

**Authors:** Claire Rochette, Nicolas Jullien, Alexandru Saveanu, Emmanuelle Caldagues, Ignacio Bergada, Debora Braslavsky, Marija Pfeifer, Rachel Reynaud, Jean-Paul Herman, Anne Barlier, Thierry Brue, Alain Enjalbert, Frederic Castinetti

**Affiliations:** 1 Aix Marseille University, CNRS UMR7286, CRN2M, Faculté de médecine, Marseille, France and Reference Center for Rare Pituitary Diseases DEFHY, La Timone Hospital, Marseille, France; 2 Laboratory of Molecular Endocrinology, La Conception Hospital, Assistance Publique Hôpitaux de Marseille, Marseille, France; 3 CHU Nantes, Department of pediatric endocrinology, Nantes, France; 4 Centro de Investigaciones Endocrinologicas (CEDIE) « Dr. César Bergada » Division de Endocrinologia, Hospital de Ninos Ricardo Guttierrez, Buenos Aires, Argentina; 5 Univ med center Ljubjana, Department Endocrinology, Zaloska 7, Ljubjana, Slovenia; University of Cordoba, SPAIN

## Abstract

LHX4 is a LIM homeodomain transcription factor involved in the early steps of pituitary ontogenesis. To date, 8 heterozygous *LHX4* mutations have been reported as responsible of combined pituitary hormone deficiency (CPHD) in Humans. We identified 4 new *LHX4* heterozygous allelic variants in patients with congenital hypopituitarism: *W204X*, *delK242*, *N271S* and *Q346R*. Our objective was to determine the role of LHX4 variants in patients’ phenotypes. Heterologous HEK293T cells were transfected with plasmids encoding for wild-type or mutant LHX4. Protein expression was analysed by Western Blot, and DNA binding by electro-mobility shift assay experiments. Target promoters of LHX4 were cotransfected with wild type or mutant LHX4 to test the transactivating abilities of each variant. Our results show that the *W204X* mutation was associated with early GH and TSH deficiencies and later onset ACTH deficiency. It led to a truncated protein unable to bind to alpha-Gsu promoter binding consensus sequence. W204X was not able to activate target promoters *in vitro*. Cotransfection experiments did not favour a dominant negative effect. In contrast, all other mutants were able to bind the promoters and led to an activation similar as that observed with wild type LHX4, suggesting that they were likely polymorphisms. To conclude, our study underlines the need for functional in vitro studies to ascertain the role of rare allelic variants of LHX4 in disease phenotypes. It supports the causative role of the *W204X* mutation in CPHD and adds up childhood onset ACTH deficiency to the clinical spectrum of the various phenotypes related to *LHX4* mutations.

## Introduction

Pituitary ontogenesis requires a precise temporal and spatial regulation of several early and late-acting transcription factors, such as LIM (LIN-11, Isl1 and MEC-3) domain transcription factors (LHX4, LHX3, LHX2 or ISL1)[1, 2]. In Humans, mutations of some of the genes coding for these transcription factors lead to combined pituitary hormone deficiency (CPHD)[3]. CPHD is a rare condition defined by the presence of at least two anterior pituitary hormone lineage deficiencies, associated with variable pituitary and extra-pituitary anomalies on MRI.

Lhx4 is specifically involved in the early steps of pituitary ontogenesis[4–6]. Although the roles of Lhx4 might be redundant at some developmental stages with those of Lhx3 (4)(6), proper expression of Lhx4 is crucial not only for the development of the rudimentary Rathke’s pouch into the mature pituitary, but also for the development of other organs such as the lungs. Murine models indeed showed that *Lhx4* homozygous inactivation (*lhx4* ^*-/-*^) led to an early death due to pulmonary failure: mice presented hypoplastic Rathke’s pouch with a decreased number of the precursors of the five anterior pituitary lineages. In contrast, mice with *Lhx4* heterozygous inactivation (*lhx4* ^*+/-*^) had a normal phenotype[7].

In humans, *LHX4* mutations are rare[8, 9]. In our cohort of patients with CPHD, only 3 mutations have been reported among the 321 patients screened to date in view of compatible phenotypic profiles (unpublished data). Since the first reported *LHX4* mutation, advances have been made on the phenotypic characterization and outcome of patients carrying *LHX4* mutations. We and others have for instance emphasized the large intra- and inter- familial variability of the phenotype. Precise profile of patients to be screened for mutations of *LHX4* remains difficult to determine, as only eight *LHX4* mutations transmitted as an autosomal dominant trait have been reported in the literature [8, 10–14]. Patients were presenting with CPHD, but the number and nature of deficient pituitary lineages were variable; extra-pituitary anomalies could be absent or include ectopic posterior pituitary, abnormal sella turcica shape or abnormal corpus callosum.

Four new *LHX4* allelic variants have been found in five distinct pedigrees through the GENHYPOPIT network, an international network that collected DNA samples and phenotype data from more than 1000 patients with CPHD. We report here the phenotypes of the patients, the results of the functional studies, and extend the genetic and phenotypic spectrum of *LHX4* mutations. Our results also raise the difficulties in proving the deleterious effect of unknown allelic variants.

## Materials and Methods

### Subjects

The GENHYPOPIT network was launched as a multicentric study involving both French and international pediatric and adult endocrinology centers. Screening of *LHX4* mutations was performed in patients with specific phenotypes as previously described (8). Hormonal studies and intracranial imaging were performed in all patients in each referring medical center. On MRI, malformations were systematically sought and recorded. Patients with a known postnatal cause of acquired hypopituitarism were excluded. After written informed consent was given, blood samples were collected from patients and, whenever possible, first-degree relatives. Informed written consent was obtained from the parents, caretakers or guardians on behalf of the minor/children enrolled in the study. The study was approved by the Ethics committee of the University of Aix-Marseille II (France).

### Screening for *LHX4* mutations

*LHX4* sequence was amplified from perip heral blood DNA. Genomic analysis of *LHX4* was performed by direct sequencing. The six coding exons of *LHX4* were amplified from genomic DNA using exon-flanking primers as previously described [8]. The same primers were used for sequencing using CEQ 8000 sequencer (Beckman Coulter, Fullerton, CA). According to the previously described genotyping algorithm (13), other candidate genes (*HESX1*, *OTX2*, *PROKR2*, *LHX3*, *SOX3*, *PROP1*) had previously been sequenced in the patients, and no alteration had been found in coding regions.

### *In silico* analyses

Alignment of amino acid sequences was performed with Clustal X (www.clustal.org) and Phylogene (acces.ens-lyon.fr/biotic/evolut/phylogene/accueil.htm) softwares. Poliphen 2 (http://genetics.bwh.harvard.edu/pph2), Predict SNP (http://loschmidt.chemi.muni.cz/predictsnp) and UMD-HTS (www.umd-hts.eu/WHTS9), were used to appraise the potential pathogenicity of each allelic variant.

### Plasmid constructs and mutagenesis

pcDNA3.1 LHX4-myc plasmid was a gift from S. Rhodes (Indianapolis, USA). *In vitro* site-directed mutagenesis was performed using the QuikChange Site-Directed Mutagenesis System (Stratagene cloning system, La Jolla, CA). In brief, 100-ng template DNA was incubated with Accuprime DNA polymerase (Invitrogen, France) and the sense and antisense primers containing the desired mutation. The reaction comprised 18 cycles consisting of 15 sec denaturation at 95°C, 30 sec annealing at 55°C, and 6.5 min extension at 68°C. After purification (NucleoSpin plasmid Miniprep kit, Macherey-Nagel) and amplification of the mutated plasmid the correct sequence was confirmed by sequencing. Notice that due to the truncated nature of the protein encoded by *W204X LHX4*, a specific primer design had to be considered to keep the myc C-terminal tag in frame. Mutagenesis primers used for each allelic variant are available on request.

Reporter constructs containing different gene-regulatory regions with putative LIM factor binding sites were fused to a firefly luciferase gene. These constructs included the proximal promoter regions of the human *PRL* gene (PRL-250) (a gift of J. A. Martial, Liege, Belgium), the proximal promoter regions of the human *TSHb* gene (a gift of S. Amselem, Paris, France), the proximal promoter regions of the human alpha-subunit *(*α*GSU)* gene (a gift of S. Amselem, Paris, France) and the positive autoregulatory site of the human *POU1F1* promoter gene (a gift of M. Delhase, Libe, Bruxelles, CA) (19).

### Western Blot

For Western Blot analyses of cells transfected with LHX4 expression vectors nuclear extracts were prepared, and were run on a 12% acrylamide gel (25 μg protein per lane). After transfer, nitrocellulose membrane (iBlot Gel transfert, Invitrogen) was incubated with the primary antibody (1/1000, Mouse anti-myc antibody, clone 9E10 ThermoFisher Scientific), followed by the secondary antibody (anti-mouse rabbit polyclonal antibody at 1/10 000, Santa Cruz biotechnology inc Texas, USA).

### EMSA

EMSA experiments were performed using the previously described LUEGO technique(14) with probes representing the pituitary glycoprotein basal element (PGBE) of the α*GSU* gene, labeled with fluorophore CY5 (5'-gtgccctggtctgg ggtacttagctaattaaatgtg-3 ‘; 3'-cacgggaccagaccccatgaatcgattaatttacac-5'). Fifteen μg of nuclear protein extracts were used. Supershift assay was performed with 0.5 μg of mouse anti-myc antibodies. Depending on condition, unlabeled wild-type (wild competitor) or mutated (mutated competitor, 5’-ggtacttagctaactgactgtg-3’) probes were used as competitors (50-fold higher concentration). Visualization was performed with Typhoon FLA 9000 scanner (GE Healthcare).

### Cell culture and transfection

HEK293T cells were cultured in 12-well plate and transfected as follows: 200 ng reporter constructs (TSHb, PRL-250, AlphaGsu, or POU1F1 promoter) and 200–400 ng effector construct (pcDNA3.1 empty vector or wild- type or mutant LHX4) per well were cotransfected using the liposome technique (Polyfect transfection reagent; QIAGEN, Hilden, Germany). Total DNA was kept constant with pcDNA3.1 empty vector, which also acted as a control. Transfection efficiency was determined using 15 ng pCMV-Renilla, and used to normalize the luciferase firefly values. Firefly and Renilla luciferase activity was measured 48 h after transfection. All experiments were repeated thrice, and all assay points were performed in triplicate. Results are expressed in relative units (fold increase *vs*. empty vector).

### Statistical analysis

Data points were compared using a one-tailed Student's t-test for paired samples using XLStat 2013.4.05 (Paris, France). Values were considered significantly different when p<0.05.

## Results

### Individual data of the patients bearing LHX4 allelic variants

#### Pedigree A: *Trp204X LHX4* allelic variant

The propositus was referred to an endocrinologist at the age of two years because of growth delay. TSH and GH deficiencies were diagnosed and the patient received L-thyroxin and GH replacement therapy. Corticotroph deficiency was diagnosed later, at the age of nine, as the patient was suffering from asthenia. Hydrocortisone was therefore initiated (20 mg/day). Due to the prepubertal age of the propositus, FSH/LH deficiency was not evaluated. Pituitary MRI, performed at the age of two, showed a hypoplastic pituitary, and an ectopic posterior lobe. The pituitary stalk was not visualized. No extra-pituitary malformation was found.

Genetic analyses showed that the propositus was bearing the allelic variant *p*.*W204X* in a heterozygous state. His parents, older sister and younger brother were clinically unaffected and no genetic analysis could be performed.

#### Pedigree B: *DelLys242 LHX4* allelic variant

ACTH, TSH and GH deficiencies were diagnosed at the age of one month because of a prolonged jaundice. Due to the prepubertal age of the propositus, FSH/LH and testosterone were evaluated at 2 months of age, and presented a normal pattern throughout post-natal gonadotropic surge. Pituitary MRI showed a hypoplastic pituitary with a normal pituitary stalk and posterior pituitary lobe.

Genetic analyses showed that the propositus was bearing the allelic variant *p*.*DelK242* in a heterozygous state. His mother, who did not have any pituitary deficiency and had normal pituitary MRI, was also carrying the same allelic variant. No other family member was available.

#### Pedigree C: *Asn271Ser LHX4* allelic variant

The propositus was born with cleft lip and palate. She had growth retardation since the first year of life and GH deficiency was diagnosed at 2 years. Central hypothyroidism was diagnosed at 3 years of age. Pituitary MRI showed hypoplastic anterior pituitary and an ectopic posterior lobe. The pituitary stalk was not visualized.

Genetic analyses showed that the propositus was bearing the allelic variant *p*.*N271S* in a heterozygous state. No other family member was available.

#### Pedigrees D and E: *Gln346Arg LHX4 allelic variant*

This allelic variant was found in two unrelated patients:

Pedigree D:

The patient presented complete GH deficiency at the age of six and TSH deficiency at the age of ten. He had hypospadias, toe agenesis and a short limb. Pituitary MRI showed a hypoplasic pituitary and an ectopic posterior lobe. The pituitary stalk was not visualized. Genetic analyses showed that the propositus was bearing the allelic variant *p*.*Q346R* in a heterozygous state. His parents, and younger brothers were clinically unaffected and no genetic analysis could be performed.

Pedigree E:

The patient presented TSH and GH deficiencies at the age of 10, ACTH and LH/FSH deficiencies at the age of 18. Pituitary MRI showed a hypoplastic pituitary and an ectopic posterior lobe. The pituitary stalk was not visualized.

**Fig 1:**
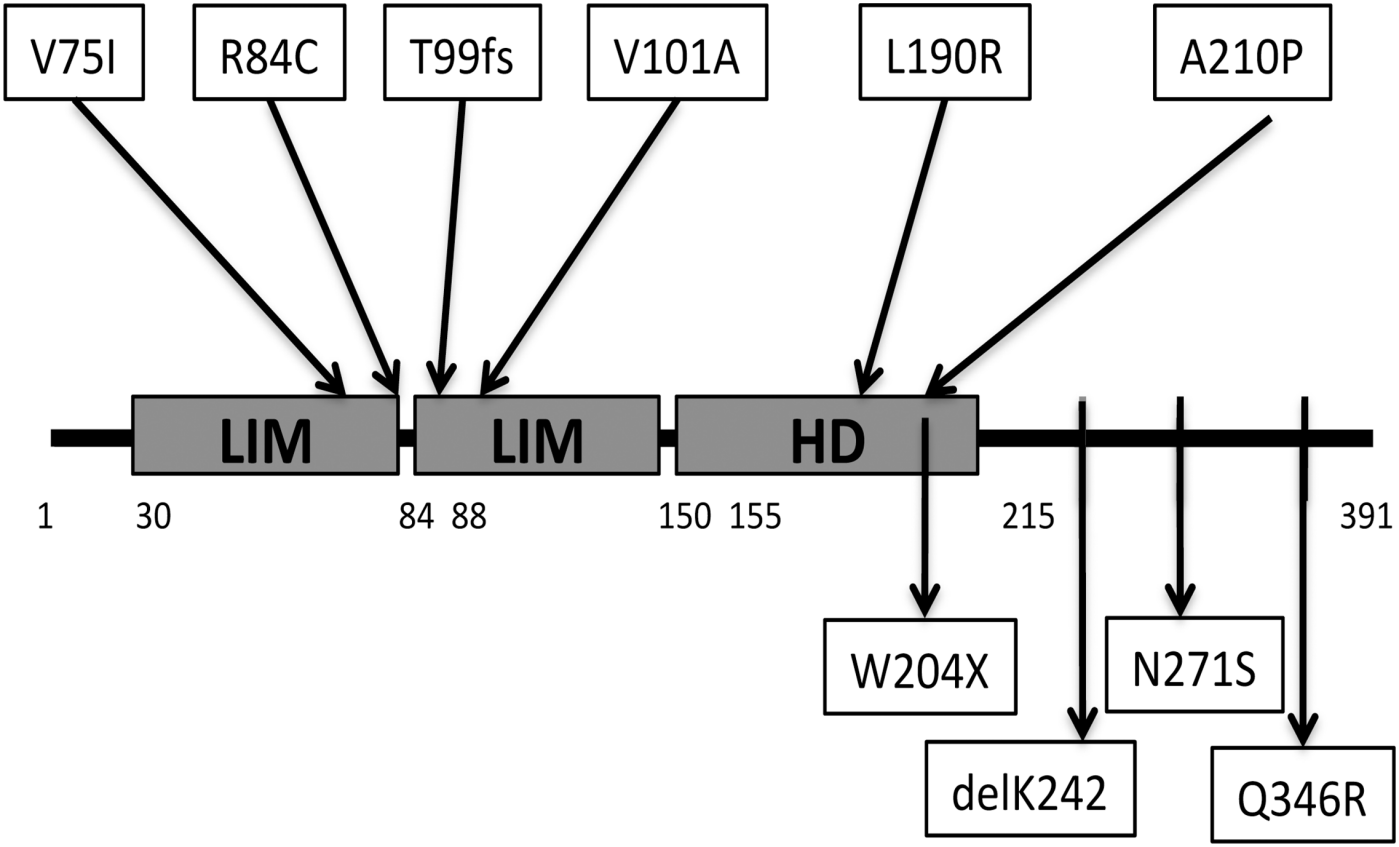
Schematic representation of *LHX4* (true length of each domain was not considered). Upper part: Previously published mutations considered pathogenic on the basis of transfection studies (9)(10)(11)(8). Note that intronic mutations are not shown(9)(12). Lower part: Newly identified allelic variants in this study. HD: homeodomain. Aminoacids 30–84, first LIM domain; aminoacids 88–150, second LIM domain; aminoacids 155–215, homeodomain (HD).

Genetic analyses showed that the propositus was bearing the allelic variant *p*.*Q346R* in a heterozygous state. No other family member was available (Table 1, Fig 1).

**Table 1:** Phenotypic and biological profiles of the patients bearing the four new allelic variants.

Mutation	W204X	DelK242	N271S	Q346R	Q346R
Year of birth	2001	2005	2003	1996	1983
Country	France	Argentina	Argentina	France	Slovenia
Medical history of hypopituitarism in family	No	No	Not specified	No	Not specified
ACTH deficiency	Yes (9 yrs)	Yes (0.1 yr)	No	NO	Yes (18 yrs)
TSH deficiency	Yes (2 yrs)	Yes (0.1 yr)	Yes (3 yrs)	Yes (10 yrs)	Yes (10 yrs)
FSH/LH deficiency	NE	NE	No	No	Yes (15 yrs)
GH deficiency	Yes (2 yrs)	Yes (0.1 yr)	Yes (2 yrs)	Yes (6 yrs)	Yes (10 yrs)
Anterior pituitary on MRI	Hypoplastic	Hypoplastic	Normal	Hypoplastic	Hypoplastic
Posterior pituitary MRI	Ectopic	Not specified	Ectopic	Ectopic	Ectopic
Pituitary stalk MRI	Not visualized	Not specified	Thin	Not visualized	Not visualized
Associated malformations	No	No	Cleft lip and palate	Hypospadias, toe agenesis, short limb	No

For pituitary deficiencies, data between brackets represent age at diagnosis. Complete GH deficiency was defined as GH response after stimulation below 10 mUI/liter. Corticotroph deficiency was defined as plasma cortisol value below 500 nmol/liter after insulin test stimulation. Gonadotroph axis was investigated only in patients of postpubertal age, *ie*. older than 15 years for female and 17 years in male subjects. FSH-LH deficiency was diagnosed on the basis of delayed or absent pubertal development with low serum testosterone or estradiol levels and blunted LH/FSH response to a GnRH stimulation test. Thyrotroph deficiency was defined as low or normal basal TSH levels associated with low T4 levels. NE, not evaluated.

### *In silico* analyses

All of the modified LHX4 amino acid residues were conserved in known LHX4 sequences in mammalians and chicken (data not shown). Prediction softwares showed that only the *W204X* allelic variant was a likely pathogenic mutation, whereas *DelK242*, *N271S*, and *Q346R* allelic variants were considered as polymorphisms.

### *In vitro* analyses

#### Western Blot

**Fig 2:**
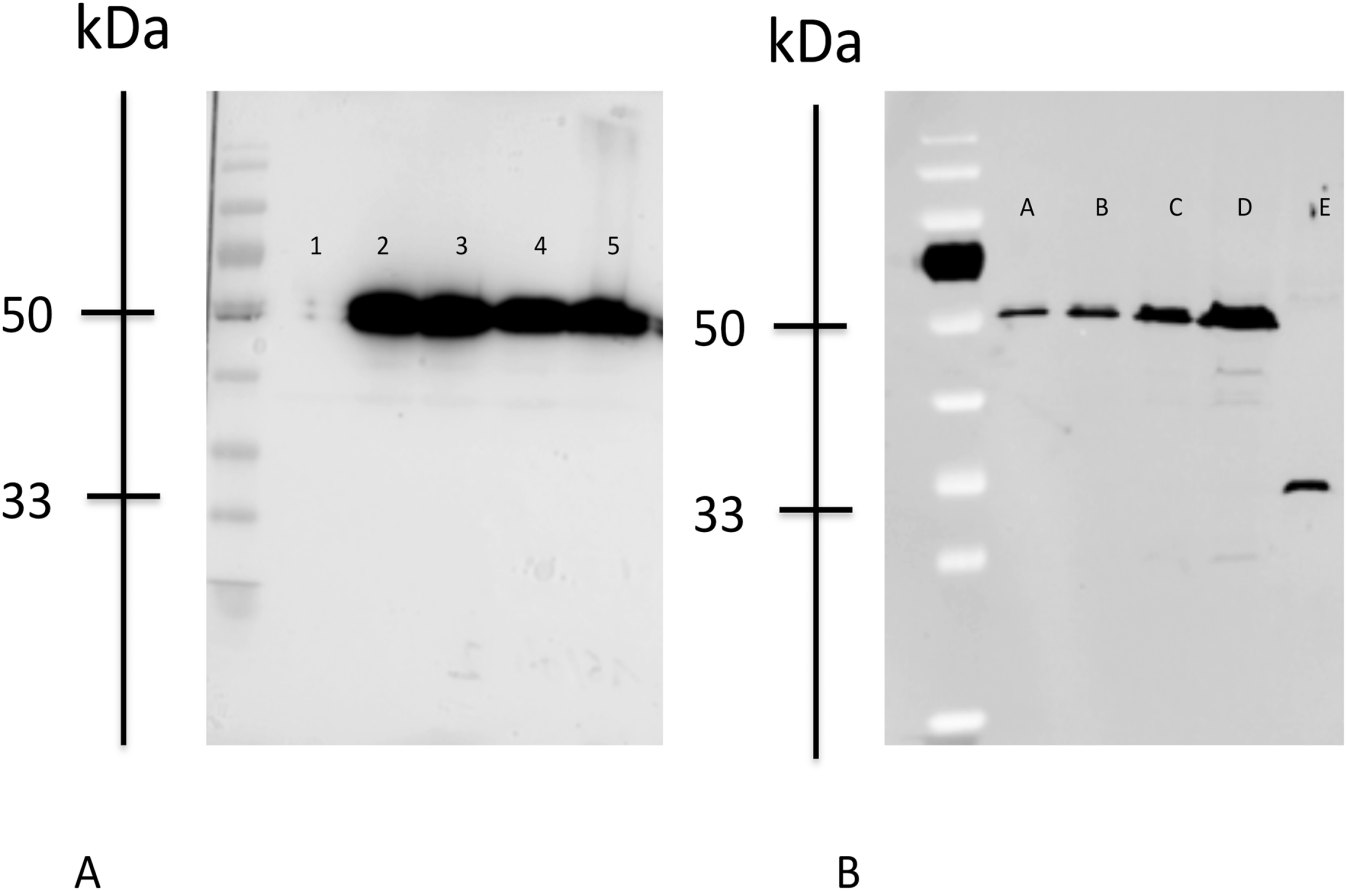
Western Blot. Western blot analysis of wild-type and mutant LHX4 proteins from transfected heterologous human embryonic kidney 293T cells. The migration positions of protein standards (kDa) are shown. Control is transfection with empty vector. Lanes are identified as follows: Fig. A; 1, pcDNA3.1 (empty vector); 2, LHX4 WT-myc; 3, DelK242-myc; 4, N271S_myc; 5, Q346R-myc; Fig. B; A: LHX4 WT-myc diluted to 1/8; B: LHX4 WT-myc diluted to ¼; C: LHX4 WT-myc diluted to ½; D: LHX4 WT-myc; E: LHX4 203-myc.

When expressed in HEK 293T cells, wild-type and DelK242, N271S, and Q346R LHX4 proteins of similar sizes (50 kDa) were detected by Western blotting (Fig 2A). As expected, the truncated W204X protein was identified with a specific 33 kDa band. Our results also suggest that the W204X protein could be under-expressed in comparison with wild-type LHX4 (Fig 2B).

#### Electro mobility shift assay

**Fig 3:**
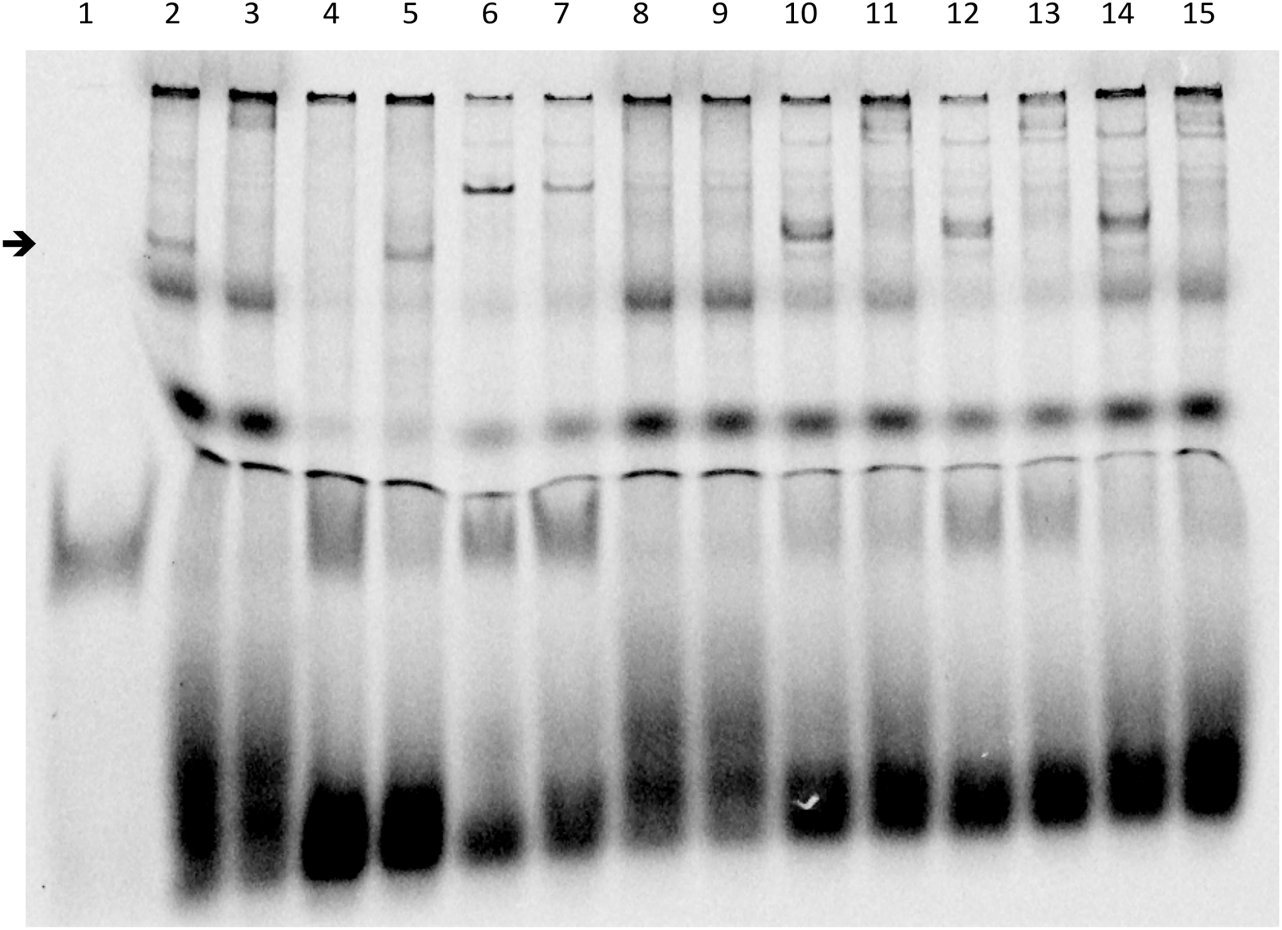
Electro Mobility Shift Assay Experiments. EMSA was performed with the alphaGSU-luego2Cy5 probe, corresponding to a consensus binding sequence of LHX4 identified on the alphaGSU murine promoter (5'-gtgccctggtctgg ggtacttagctaattaaatgtg-3 ‘; 3'-cacgggaccagacc-ccatgaatcgattaatttacac-5'). Supershift was performed with a monoclonal anti-myc antibody. Arrow indicates position of the expected LHX4 DNA binding. Lanes are identified as follows: 1, free probe; 2, LHX4 wt (band corresponding to LHX4 identified by the arrow); 3, LHX4 wt + anti-myc antibody (supershift, no LHX4 band visualized at the arrow level); 4, non mutated competitor (LHX4 band is not visualized, as the probe is bound by the competitor); 5, mutated competitor (LHX4 band is visualized at the arrow level as the competitor can not bind the probe); 6, PcDNA3.1 (empty vector); 7, PcDNA3.1 + anti-myc antibody; 8, W204X; 9, W204X + anti-myc antibody (supershift); 10, DelK242; 11, DelK242 + anti-myc antibody (supershift); 12, N271S; 13, N271S + anti-myc antibody (supershift); 14, Q346R; 15, Q346R + anti-myc antibody (supershift).

To test the DNA binding properties of the altered LHX4 proteins, we performed EMSA using probes representing the α*GSU* gene promoter. The delK242, N271S and Q346R proteins, which have an intact homeodomain, bound with similar efficiency as the wild-type protein. By contrast, and consistent with the lack of a part of the homeodomain, the W204X protein did not bind to the cognate probe (Fig 3).

#### Transfections

**Fig 4:**
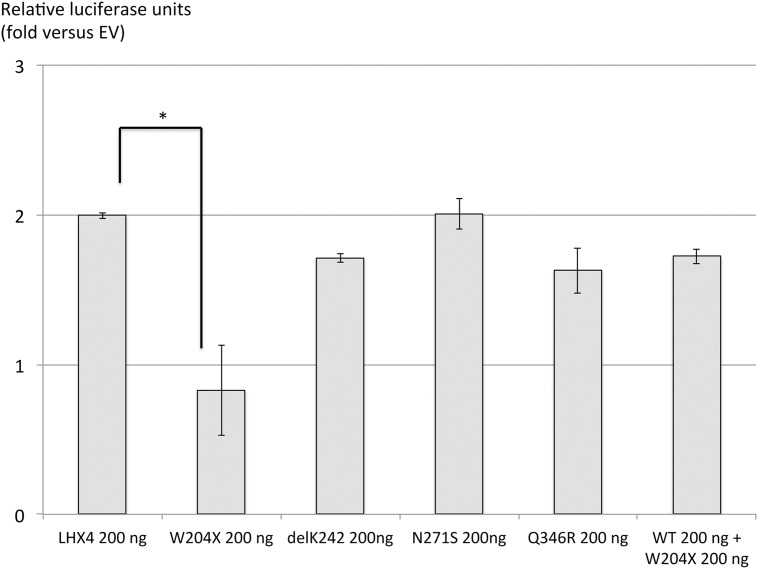
Expression vectors for wild-type (WT) and the four allelic variants with bTSH promoter. Plasmids expressing the various LHX4 mutants were transiently cotransfected into heterologous HEK293T cells with a luciferase reporter gene under the control of the bTSH promoter. Promoter activity was assayed by measuring luciferase activity 48h after transfection. Negative controls (control) received equivalent amounts of empty expression vector plasmid. Results are expressed as fold-increase of luciferase signal relative to control. *p<0.05.

Cotransfection of wild-type *LHX4* with *TSHb* promoter resulted in a strong stimulation of the luciferase reporter gene relative to the empty vector. No activation was observed with the *W204X* mutant. Cotransfection of mutant *W204X* and equivalent amounts of wild-type LHX4 did not modify the effect of the latter on TSHb promoter, arguing against a dominant negative effect. In contrast, cotransfection of *DelK242*, *N271S* or *Q346R LHX4* allelic variants with the *TSHb* promoter resulted in the stimulation of luciferase activity similar to that evoked by wild-type *LHX4* (Fig 4). The same results were observed for the activation of the alphaGsu, POU1F1 and prolactin promoters by the four allelic variants (data not shown). Taken together these results suggest that *DelK242*, *N271S* and *Q346R LHX4* were probably non-deleterious polymorphisms, whereas *W204X* was a defective mutation.

## Discussion and Conclusion

*W204X* mutation represents a new pathogenic allelic variant of *LHX4* found in a patient presenting with combined pituitary hormone deficiency. Identification of *LHX4* mutations linked to such defects have rarely been reported in the literature (8 mutations reported to date) [8–14] and in our large GENHYPOPIT cohort of patients with CPHD (3 mutations in 321 patients screened, unpublished data). This rarity may, at least in part, be due to the fact that homozygous *LHX4* mutations are likely lethal in Humans: this is suggested by the fact that patients bearing *LHX4* mutations were always reported as heterozygous, and that mice with homozygous inactivation of *Lhx4* died shortly after birth[7]. Comparison with the murine model is however difficult, as mice with heterozygous inactivation of *Lhx4* are unaffected[7].

The mechanism by which *W204X* mutation leads to a functionally defective protein is an abnormal DNA binding, as shown by the results of EMSA experiments. The functional alteration induced by this new mutation could also have been due to nonsense-mediated RNA decay. However, the mutant protein is expressed in our cells, as shown by Western blot results, argueing against this hypothesis. Abnormal DNA binding could be expected as the protein lacks a part of the homeodomain. We had previously reported such a mechanism for the *Thr99fs LHX4* mutation (8), but the predicted truncated protein was lacking the whole homeodomain. Our results suggest that LHX4 amino acids 204–215, which encode for the third DNA-recognizing alpha-helix of the homeodomain, are crucial for proper DNA binding. Consistent with our results, Pfaeffle *et al*. had previously shown that a point mutation in this area (*A210P*) also led to abnormal DNA binding[8].

Delayed corticotroph deficiency (that appeared by the age of 9) is the main striking phenotypic feature of our patient bearing *W204X* mutation. This delayed deficiency had been reported at age 16 in another patient carrying *LHX4 R84X* mutation[14]. It is surprising as the other patients with *LHX4* mutations had early dysfunction of pituitary lineages. However, the father of the propositus carrying *Thr99fs* mutation had normal size, as did 2 children carrying the same mutation, despite GH and LH/FSH deficiency diagnosed by the age of 40 [8]. This suggested that delayed onset deficiencies can be observed in patients with *LHX4* mutations. Delayed corticotroph, GH and TSH deficiencies have already been reported in patients carrying mutations of *PROP1*, a paired homeodomain transcription factor, involved in later stages of pituitary development [15, 16]. For these 2 transcription factors, the mechanisms of delayed deficiency remain however unknown [4].

Our functional studies did not show any deleterious effect of the three allelic variants *DelK242*, *N271S and Q346R*. Despite the fact that these variants have not been identified in SNP databases (data not shown), and that the patients were presenting with phenotypes compatible with *LHX4* mutations, our results supported their classification as rare polymorphisms (*ie*. they were not responsible for the phenotype of CPHD). Of note, the delK242 variant has been found in a heterozygous state in 3 individuals (3 out of 6259, i.e., 0.05%) from an American population, (see Exome Variant Server website URL: http://evs.gs.washington.edu/EVS/). Other genes coding for transcription factors have been screened, but no clear etiology could be identified. Other as yet unknown transcription factors or pathways might be involved in the phenotypes. Environmental causes during pregnancy may also be involved. Another possibility could be that our functional studies, performed in heterologous cells (chosen as they were not expressing endogenous LHX4), in an in vitro system, and on a limited number of target genes, did not manage to show a true defective mechanism due, for instance, to the lack of the cofactors network found in pituitary cells. These results however are consistent with the fact that the 3 amino-acids were located in non-functional domains, and that in silico analysis was in favour of silent polymorphisms. Nevertheless, defective mutations in supposedly non-functioning domains have already been reported for other transcription factors: for instance, LHX3 lacking a small part of its C-terminus domain (*LHX3 W224X*) has been shown to be functionally defective. It can be noted also that *in silico* analysis is not always reliable: *LHX4 V101A* and *A210P* mutants had indeed been considered as silent polymorphisms by prediction software, whereas functional studies showed that they had decreased transactivating abilities on pituitary hormone promoters [11, 13]. Analysing the potential pathogenic status of allelic variants by *in silico* analysis and on the basis of *in vitro* functional studies clearly suggests the need for a global approach of such new allelic variants, including both approaches, as well as disease segregation in the family: this last parameter might also be misleading, as shown by the example of the father of the propositus carrying *c*.*293InsC* mutation who had a normal gonadotroph axis, allowing him to transmit the mutation to his 2 affected children, and who finally had delayed gonadotroph deficiency at the time of molecular diagnosis [8]. All these points clearly show the difficulty in determining the pathogenic nature of some variants. Moreover identifying a patient with a presumed polymorphism does not rule out the need for a close monitoring over time to allow for timely diagnosis and treatment of pituitary hormone deficits of possibly delayed onset.

To conclude, we report here four new allelic variants of *LHX4*, including one unequivocally deleterious mutation responsible for an unusual CPHD phenotype with delayed corticotroph deficiency. The fact that the 3 other variants were identified in patients with phenotypes compatible with *LHX4* mutations emphasizes the difficulties in determining the pathogenicity of such variants, using *in vitro* functional studies. Indeed, some uncertainty remains as to the accuracy of such studies to predict the possible involvement of such rare allelic variations in the observed phenotype. Identifying new etiologies of CPHD should allow improving the management of patients with yet unknown clear explanation of their phenotype.
